# 肿瘤微环境——肿瘤转移的关键因素

**DOI:** 10.3779/j.issn.1009-3419.2015.01.08

**Published:** 2015-01-20

**Affiliations:** 150081 哈尔滨，哈尔滨医科大学附属肿瘤医院肿瘤内科 Department of Medical Oncology, Tumor Hospital, Harbin Medical University, Harbin 150081, China

**Keywords:** 肿瘤微环境, 肿瘤转移, 分子机制, Tumor microenvironment, Tumor metastasis, Molecular mechanism

## Abstract

肿瘤转移是癌症治疗失败和患者死亡的主要原因，其分子机制复杂，涉及多步骤、多阶段、多基因的变化。作为肿瘤细胞赖以生存的场所，肿瘤微环境在肿瘤转移过程中起到至关重要的作用。因此，研究肿瘤微环境与肿瘤转移的动态关系，阐明微环境中不同因子在转移过程中的分子机制是抑制肿瘤转移的关键。

## Abstract (en)

Metastasis is the main reason for the failure of cancer therapy and the death of patients. The molecular mechanism is complex, involving the change of multi-step, multi-stage and multi-gene. As the survival places of tumor cells, the tumor microenvironment plays a crucial role in tumor metastasis. Therefore, the study of the dynamic relationship between the tumor microenvironment and tumor metastasis, to clarify the molecular mechanisms in the metastasis process is the key point to inhibit the tumor metastasis.

目肿瘤转移是肿瘤研究工作的困境之一，超过90%的癌症患者死于肿瘤转移^[1]^。恶性肿瘤细胞的转移是区别正常细胞的特征之一，不同肿瘤细胞转移能力不同，而同一种肿瘤细胞也存在具有不同转移潜能的亚群，同时还存在转移的器官特异性。由此可见，肿瘤转移是一个极其复杂的过程，涉及多方面因素，不但包括肿瘤细胞本身的特征变化，还包括宿主环境的变化。肿瘤微环境是一个动态网络，它包括肿瘤细胞、细胞外基质和间质组织等，是影响肿瘤转移的关键因素^[2]^。深入研究肿瘤微环境影响转移的分子机制，早期识别微环境中转移相关因子的改变可为肿瘤转移的早期诊断及治疗提供新思路。肿瘤转移不但涉及原发肿瘤部位微环境的改变，而且还包括肿瘤细胞对继发转移部位微环境的适应，本文将从这两方面对近年来微环境中转移相关因子的研究新进展进行综述。

## 1 原发肿瘤部位相关微环境

目前认为肿瘤转移可大致分为两个部分，首先是原发肿瘤部位细胞增殖、新生血管生成，肿瘤细胞穿破细胞外基质屏障向外侵袭，随后肿瘤细胞在继发部位增殖形成实体瘤。原发肿瘤部位微环境主要包括肿瘤相关巨噬细胞、肿瘤相关纤维细胞、髓源性抑制细胞、肥大细胞等，这些细胞可分泌多种细胞因子、趋化因子促进肿瘤转移。特定的肿瘤微环境可以通过影响肿瘤细胞的增殖、调节转移相关基因的表达水平、诱导血管生成、促进细胞外基质的降解从而促进转移的发生。

### 1.1 肿瘤相关巨噬细胞（tumor-associated macrophages, TAMs）

TAMs属于M2d亚型巨噬细胞，起源于循环血液单核细胞或组织中的巨噬细胞，是肿瘤微环境的重要组成成分，在肿瘤进展及转移的各个步骤中起关键作用^[3]^。早期侵袭转移阶段，肿瘤细胞释放趋化因子吸引巨噬细胞和其他炎症细胞到达肿瘤周围的基质区域，随后TAMs可穿透基底膜，从而使肿瘤细胞逃离基底膜的束缚到达周围正常组织基质，同时TAMs和肿瘤细胞均可刺激血管生成，提高细胞的侵袭性和运动性^[4]^。TAMs可通过释放血管生成调节酶类，如基质金属蛋白酶-2（matrix metalloprotein 2, MMP2）、MMP-7、MMP-9、MMP-12和环氧合酶2（cyclooxygenase 2, COX2）等促进新生血管形成，新生血管可为肿瘤生长提供营养和氧分，并为肿瘤细胞的转移提供路径。TAMs还可释放一系列直接促进肿瘤细胞浸润和转移的细胞因子及生长因子，如血管内皮生长因子（vascular endothelial growth factor, VEGF）、肿瘤坏死因子α（tumor necrosis factor, TNF-α）、白介素8（interleukin 8, IL-8）和碱性成纤维细胞生长因子（basic fibroblast growth factor, bFGF）等。缺氧环境下TAMs上调表达缺氧诱导因子1（hypoxia inducible factor-1α, HIF1）和HIF2，HIF活化可以诱导血管新生促进转移^[3, 5]^。

近年来TAMs分化的分子机制研究更加深入，有研究^[6]^表明乳腺癌中TAMs表面表达血管内皮细胞粘附分子1（vascular cell adhesion molecular 1, Vcam-1），Vcam1可介导肿瘤细胞与基质细胞间的相互粘附，并且TAMs的分化依赖于Notch信号通路及关键转录因子RBPJ的调控，去除TAMs可恢复细胞毒性T细胞应答从而抑制肿瘤生长，而乳腺组织中的巨噬细胞却没有此作用。乳腺癌缺氧组织区域中嗜酸性粒细胞趋化因子（Eotaxin）和抑瘤素M（oncostain M）高表达，可以促进巨噬细胞分化为M2型TAMs，抑制Eotaxin和Oncostain M的表达不但可以抑制M2型TAMs在缺氧区域聚集还可以增强贝伐单抗的抗血管生成效应^[7]^。前列腺癌中肾母细胞瘤过表达基因（nephroblastoma overexpressed, NOV/CCN3）表达上调，NOV/CCN3可以招募巨噬细胞并促进其分化为M2型TAMs，其机制是M2型TAMs中NOV/CCN3可激活粘着斑激酶（focal adhesion kinase, FAK）/Akt/NF-kB信号通路导致VEGF表达增加从而促进肿瘤血管生成^[8]^。Wen等^[9]^在卵巢癌缺氧环境下研究发现5-脂氧合酶（5-lipoxygenase, 5-LOX）代谢物表达增加可以促进TAMs的浸润，其机制是通过p38信号通路上调TAMs中MMP-7的表达，增加TNFα和肝素结合性表皮生长因子的释放，从而促进TAMs的转移和侵袭。动物模型中选择性5-LOX抑制剂可降低MMP7的表达和TAMs的数量。Yang等^[10]^研究还发现MicroRNA-19a-3p具有调节TAMs的作用，MicroRNA-19a-3p在4T1小鼠乳腺癌TAMs中表达下调，同时伴有促进乳腺癌侵袭转移的Fos相关抗原1基因（Fos-related antigen 1, Fra-1）表达上调。上调MicroRNA-19a-3p的表达可减少VEGF、信号转导及转录激活因子3（signal transducer and activator of transcription 3, STAT3）和磷酸化STAT3的表达，细胞侵袭转移能力下降。这也为MicroRNA在TAMs与转移相关的研究中奠定了理论基础。分子影像学的发展使活体状态下细胞和分子水平的定性及定量研究成为现实，Lohela等^[11]^应用活体共聚焦显微镜动态观察小鼠乳腺肿瘤中清除TAMs和树突状细胞亚群的状况，从全新的角度加深了对肿瘤微环境的认识。这些都为清除TAMs的治疗提供了新思路。

### 1.2 肿瘤相关纤维细胞（cancer-associated fibroblasts, CAFs）

肿瘤相关成纤维细胞存在于肿瘤间质中，通过促进上皮间质转化和参与肿瘤血管生成促进肿瘤的侵袭和转移。CAFs促进肿瘤生长和转移还可继发于活性氧诱导的代谢应激反应，基质中的成纤维细胞通过HIF1α和NF-kB信号通路发生氧化应激、自噬、糖酵解，这些分解代谢的CAFs为肿瘤的生长创造了营养丰富的微环境^[12]^。CAFs可以产生多种细胞因子、细胞外基质蛋白和蛋白水解酶类物质，包括基质细胞衍生因子1（stromal cell-derived factor 1, SDF1）、肝细胞生长因子（hepatocyte growth factor, HGF）、VEGF、血小板衍生生长因子受体（platelet-derived growth factor, PDGF）、转化生长因子β（transforming growth factor β, TGFβ）、胶原蛋白和纤维连接蛋白等^[13, 14]^。PDGF和TGFβ信号通路是CAFs经典下游信号通路。最新研究表明肿瘤基质CAFs过表达PDGF受体，与易转移和预后差密切相关。PDGF可以刺激CAFs中糖蛋白斯钙素1（stanniocalcin-1, STC1）表达上调，原位结肠癌小鼠模型中抑制CAFs中STC1的表达可减少肿瘤细胞进入血管，抑制细胞的上皮间质转化从而减少远处转移的发生^[15]^。提示以PDGF信号通路中关键分子为靶点的治疗可能为抑制CAFs提供新途径。Ting等^[16]^将前列腺基质细胞暴露于前列腺癌细胞条件培养基中，观察到基质细胞的侵袭性增加，α平滑肌肌动蛋白和波形蛋白表达增加，成纤维细胞分化成为CAFs表型，此过程依赖TGFβ2信号通路，给予水飞蓟宾后可以直接抑制α平滑肌肌动蛋白的表达和CAFs形成，提示水飞蓟宾可能通过靶向CAFs起到预防前列腺癌进展的作用。CAFs还可通过其他通路促进转移的发生，表达趋化因子14[chemokine（C-X-C motif）ligand 14, CXCL14]的CAFs中一氧化氮合成酶1（nitric oxide synthase 1, NOS1）表达上调，从而激活NRF2（NF-E2 related factor 2, NRF2）和HIF1α信号通路。NRF2信号传导通路是机体抵抗内外界氧化和化学刺激的防御性传导通路，它具有双重作用，在正常组织中可以抑制肿瘤的形成，而在肿瘤组织促进肿瘤细胞增殖。HIF1α信号通路是缺氧环境下促进血管新生的重要途径。下调NOS1的表达可减少巨噬细胞的浸润，抑制表达CXCL14的CAFs生长，从而抑制转移的发生^[17]^。提示CAFs可以作为肿瘤转移治疗中的有效靶点。

### 1.3 髓源性抑制细胞（myeloid-derived suppressor cells, MDSCs）

MDSCs是一类具有免疫抑制功能的细胞群体，包括骨髓祖细胞和不成熟的髓样细胞，可抑制T细胞反应。肿瘤细胞产生的多种细胞因子均可诱导MDSCs增殖，如COX2、IL-6、粒细胞巨噬细胞集落刺激因子（granulocyte macrophage colony stimulating factor, GM-CSF）、VEGF等^[18]^。肿瘤组织过表达COX2可产生大量的前列腺素E2（prostaglandin E2, PGE2），介导MDSCs的免疫抑制作用。体外实验证实PGE2与其受体亚型EP2和EP4结合可激活p38MAPK/ERK信号通路，导致TGFβ分泌增加，单核细胞MDSCs即通过TGFβ通路抑制NK细胞的活性；沉默COX2后小鼠脾脏中CD11b^+^Gr1^+^MDSCs聚集减少，NK细胞活性增加，提示避免MDSCs的免疫抑制作用可能提高宿主的抗肿瘤反应^[19]^。Oh等^[20]^在小鼠乳腺癌动物模型研究中发现MDSCs产生的IL-6及IL-6可溶性受体α（soluble interleukin 6 receptor α, Sil-6Rα）可持续激活STAT3，通过IL-6转化信号通路促进乳腺癌细胞的侵袭。高浓度的GM-CSF可导致MDSCs的累积，敲除转录因子KLF4可通过CXCL5/趋化因子受体2[chemokine(C-X-C motif) receptor 2, CXCR2]途径降低循环血中GM-CSF的浓度，减少骨髓、脾、原发肿瘤灶中MDSCs的聚集，延缓肿瘤发展，抑制肺转移灶的形成^[21]^。溶酶体酸脂酶（lysosomal acid lipase, LAL）缺乏可导致MDSCs在多个器官中浸润并参与炎症反应，提示LAL在调节MDSCs刺激肿瘤细胞增殖方面起重要作用，研究^[22]^还发现MDSCs可分泌IL-1β和TNFα，通过激活mTOR信号通路促进肿瘤细胞的增殖。MicroRNA在调节MDSCs分化及表达过程中也起到重要作用，Wang等^[23]^在黑色素瘤及肺癌小鼠移植瘤模型中发现敲除MicroRNA155可促进肿瘤中MDSCs的聚集，导致细胞因子释放增多且肿瘤转移能力增强，提示上调MicroRNA155可能抑制MDSCs的促肿瘤转移作用。以上研究表明MDSCs在肿瘤组织中的聚集与转移密切相关，中和可诱导产生MDSCs的介质或抑制肿瘤组织对MDSCs的招募有待进一步研究。

### 1.4 肥大细胞（mast cells, MC）

MC是一种人体中广泛分布的免疫细胞，来源于骨髓造血祖细胞，刚进入外周血时处于未成熟状态，当进入外周组织后成熟并定居^[24]^。生理状态下它参与组织重塑、伤口修复，病理状态下MC分泌多种生物活性物质，如组胺、白三烯参与机体过敏反应，还能够释放多种生长因子如FGF-2、VEGF、TGF-β等促进肿瘤血管生成，影响肿瘤的侵袭和转移^[25]^。

在Snail过表达且具有*KARS*突变的胰腺导管腺癌小鼠模型中MC数目增加，干细胞生长因子（stem cell factor, SCF）表达增加，同时发现在人原发胰腺导管腺癌肿瘤组织中Snail过表达也与MC浸润增加相关，并且与SCF的表达呈正相关^[26]^。另有研究^[27]^证实表达SCF的肿瘤组织可以招募MC至原发肿瘤部位，通过释放MMP9、VEGF等调节因子促进肿瘤早期阶段的发展，在此过程中KIT下游信号通路FES酪氨酸蛋白激酶起关键作用。

MC还可与MDSCs相互作用，增加MDSCs的免疫抑制功能，协同增加细胞因子IL-6、IL-13、TNF-α和巨噬细胞炎性蛋白1α（macrophage inflammatory protein 1α, MIP-1α）的产生^[28]^。Martin等^[29]^研究发现MDSCs表面表达组胺受体（histamine receptor, HR）1、HR2和HR3，MC释放的组胺与MDSCs表面HR结合后可使MDSCs过表达精氨酸酶1（Arginase 1, Arg1）和诱导型一氧化氮合酶iNOS，增加IL-4和IL-13表达，从而促进肿瘤转移，应用HR1或HR2拮抗剂可阻断组胺介导的MDSCs增殖作用。同时还发现MC对MDSCs的转运也十分重要，在MC缺乏的鼠模型中并未观察到MDSCs转运到肝脏的现象。由此可见肥大细胞和其所释放的组胺参与了MDSCs的免疫调节过程。

## 2 转移瘤部位微环境

Steven Paget关于肿瘤转移的“土壤和种子”理论是肿瘤研究史上的里程碑事件。肿瘤细胞在转移部位的增殖是转移瘤形成的基础条件，转移靶器官的微环境决定了转移灶最终能否形成。肺、肝、骨、脑是最常见的肿瘤转移靶器官，不同靶器官可释放不同的细胞因子招募肿瘤细胞，并促进细胞增殖、诱导血管生成，最终形成转移灶。在此过程中肿瘤细胞也可通过旁分泌等方式释放有利于自身生存的各种细胞因子塑造合适的微环境定居。因此，肿瘤细胞与靶器官微环境之间的相互作用完成了复杂的转移过程，二者缺一不可。

### 2.1 肺转移灶微环境

肺组织是肿瘤转移最常见的靶器官，这可能与肺组织的解剖结构和生物学特性密切相关。肺毛细血管密集含氧丰富，可为肿瘤细胞的增殖提供便利条件。大量的肺泡巨噬细胞还可以分泌多种细胞因子，促进细胞运动、侵袭及新生血管生成。

#### 2.1.1 肺转移灶细胞外基质

肺转移灶的形成与细胞外基质成分，如巨噬细胞，MDSCs等密切相关。Qian等^[30]^在乳腺癌肺转移模型中用三种不同的方法清除巨噬细胞，结果发现清除巨噬细胞不但影响肺部转移瘤的生长，而且影响转移肿瘤细胞在肺部的种植，因此得出结论，巨噬细胞在肺转移灶的形成和生长中是必需的。肝细胞癌肺转移的研究中发现肺部微环境还受干扰素α的调节，干扰素α（interferon α, IFN-α）可以通过抑制肺部巨噬细胞的浸润，下调MMP9的表达从而调节肺部微环境抑制肺转移灶的形成^[31]^。转录因子Nrf2也可以通过调节MDSCs影响肺转移的形成，Satoh等^[32]^研究发现缺乏Nrf2的小鼠肺癌模型更容易发生肺转移，其机制是由于敲除Nrf2导致MDSCs增加，MDSCs可以利用活性氧簇（reactive oxygen species, ROS）改变T细胞受体复合物从而减弱CD81T细胞免疫效应。

#### 2.1.2 转移瘤细胞携带原发灶“土壤”

除了细胞外基质的变化影响肿瘤细胞的种植，循环血液中的肿瘤细胞也可以携带原发瘤部位的基质成分转移到肺部，包括激活的成纤维细胞等。研究表明当转移的肿瘤细胞伴随肿瘤基质细胞碎片进入循环血液时，肿瘤细胞的存活能力更强，可以为它们在肺部的种植和初期生长提供有利环境。当CAFs随肿瘤细胞进入转移部位时可以通过分泌VEGF促进肿瘤血管生成，清除部分CAFs后可减少肺转移灶数目^[33]^。

### 2.2 肝转移灶微环境

肝脏是许多恶性肿瘤转移的靶器官，这与肝脏的特殊结构密切相关，肝脏血流丰富，具有双重血供，含有众多的免疫细胞成分。转移的肿瘤细胞通过血液循环到达肝脏与肝血窦的微环境相互作用。肝血窦细胞具有双重作用，既可以抑制肿瘤细胞定居，也可以有利于它们的存活和生长，肿瘤细胞的最终结局主要受粘附分子、骨髓衍生细胞和趋化因子的影响。

#### 2.2.1 肝血窦

肿瘤细胞在经过肝血窦血管时受到机械应力发生变形，成团的肿瘤细胞在阻塞肝血窦血管时可以导致血流受阻发生短暂的缺血触发炎症反应，肝窦内皮细胞（hepatic sinusoidal endothelial cells, HSEC）释放NO和IFN-γ上调肿瘤细胞内的FasL，局部的kupffer细胞还释放多种细胞因子（IL-6、IL-8、TNF-α）和趋化因子（单核细胞趋化蛋白1、MIP-1α、MIP-2）活化NK细胞起到杀伤作用，这些因素均可以使肿瘤细胞受损或死亡。而另一方面肝血窦的炎症也可以促进肿瘤细胞粘附在血管内皮细胞，使肿瘤细胞逃离kupffer细胞和NK细胞的杀伤。这其中细胞表面粘附分子E-选择素、VCAM-1、细胞间粘附分子1（intercellular cell adhesion molecule-1, ICAM-1）和癌胚抗原（carcino enbryonic antigen, CEA）起重要作用，它们使肿瘤细胞在肝血窦中达到稳定状态。转移早期形成阶段肝血窦的中性粒细胞数量明显增加，它主要是通过与肿瘤细胞结合共定位在肝血窦中，同时伴有CXCL1的表达增加，清除中性粒细胞后肝脏肿瘤细胞的种植明显减少。提示中性粒细胞募集肿瘤细胞的作用在肝转移形成中十分重要^[34]^。

#### 2.2.2 趋化因子和趋化因子受体

研究^[35]^表明多种趋化因子和趋化因子受体在肝转移形成中起促进作用，如下调肿瘤基质和造血细胞CC类趋化因子受体1（C-C motif chemokine receptor-2, CCR1）的表达可以减少肝脏中单核细胞的浸润，减缓肿瘤生长。ShRNA敲除CXCL1后可以抑制结肠癌肝转移瘤细胞的增殖和浸润，CXCL1促进肿瘤形成的机制可能是通过NF-κB和Akt信号通路^[36]^。抑制CC趋化因子2[the chemokine (C-C motif) ligand 2, CCL2]/CCR2信号通路可以减少CD11b/Gr1髓源细胞的数量，减少肿瘤负荷，因此骨髓衍生细胞在肝脏微环境中的作用也不容忽视^[37]^。CCL20/CCR6、CCR3/CXCL10等都可以促进肝转移瘤的形成。

此外，肝转移微小病灶形成后，蛋白酶降解细胞外基质和新生血管的形成可扩大肝转移灶的范围。肝脏微环境成分与肿瘤细胞相互作用最终决定转移瘤的结局。

### 2.3 骨转移灶微环境

肺癌、乳腺癌、前列腺癌及甲状腺癌发生骨转移的几率高达70%，骨转移严重影响患者的生活质量，而目前仍没有有效的治疗手段。骨微环境包括成骨细胞、破骨细胞、造血细胞和内皮细胞等，与肿瘤细胞相互作用影响转移的发生。

#### 2.3.1 溶骨性骨转移

TGF-β信号通路在乳腺癌细胞造成溶骨性破坏中起重要调节作用，最新研究表明另一条信号通路肝癌缺失基因1（deleted in liver cancer 1, DLC1）-Rho信号通路可特异性调节乳腺癌细胞在骨随中定植，Yufeng Wang等在乳腺癌小鼠模型中敲除DLC1可使骨转移增加，其机制是由于DLC1缺失导致Rho-ROCK信号通路激活，RhoGTP酶可与细胞分裂周期蛋白42（cell division cycle 42, CDC42）协同调节肿瘤细胞骨架及运动特性，还可以调节TGFβ诱导的SMAD3接头区域磷酸化从而激活甲状旁腺激素类激素（parathyroid hormone-like hormone, PTHLH）的转录，肿瘤细胞产生的PTHLH可以作用于成骨细胞，改变骨基质中核因子κB活化因子受体配体（receptor of activator of NF-κB ligand, RANKL）/正骨保护素（osteoprotegerin, OPG）的比率，诱导破骨细胞成熟导致骨破坏的发生，因此骨转移微环境中DLC1-Rho信号通路特异性调节肿瘤细胞对TGF-β信号通路的反应，从而重建溶骨性微环境为肿瘤细胞定植提供条件^[38]^。

#### 2.3.2 成骨性骨转移

前列腺癌骨转移通常为成骨性骨转移，前列腺癌细胞可以产生一系列骨形态发生蛋白（bone morphogenetic proteins, BMP），其中BMP6可以增加肿瘤的侵袭特性，动物实验中发现抑制BMP6的表达可以抑制成骨细胞活性，缩小骨内转移灶的大小，但并不能影响皮下肿瘤的大小，提示BMP6可以促进前列腺癌细胞侵入骨微环境^[39]^。

除此之外，骨转移微环境中还含有一些细胞因子和趋化因子，如PDGF、CXCL12等。三阴性乳腺癌更易发生骨转移是由于这种类型的乳腺癌肿瘤细胞Src活性更高，其PI3K-Akt信号通路易被CXCL12和胰岛素样生长因子1（insulin-like growth factor, IGF1）激活，而骨髓微环境比其他转移靶器官含有更丰富的CXCL12和IGF1从而更易吸引肿瘤细胞到达骨髓^[40]^。

### 2.4 脑转移灶微环境

40%以上的肺癌和乳腺癌患者出现脑转移，肿瘤细胞可穿过血脑屏障在脑部微环境下存活并增殖，而大部分药物难以通过血脑屏障到达脑部杀灭肿瘤细胞，这在很大程度上加大了脑转移的治疗难度。脑转移灶的形成机制复杂，包括微环境中的血管重建、血脑屏障的破坏和肿瘤细胞穿透血脑屏障后的增殖等。

#### 2.4.1 脑微环境中的血管重建

脑转移灶中血管重建主要是通过VEGF的作用，VEGF作为促血管生成因子在脑转移瘤中表达升高，它能够使血管通透性增加，使纤维蛋白溶酶等更容易进入细胞间质，还能促进内皮细胞迁移从而有助于血管重建^[41]^。

#### 2.4.2 血脑屏障的破坏

血脑屏障的破坏是由于肿瘤细胞影响了临近内皮细胞结构和功能，三阴性乳腺癌中大脑微血管内皮细胞（brain microvascular endothelial cells, BMEC)过表达血管生成素2（angiopoietin-2, Ang-2），它可以改变紧密连接蛋白ZO-1和紧密连接蛋白claudin-5的结构从而增加血脑屏障的通透性。应用Ang2的中和抗体可以逆转BMEC的不稳定，防止血脑屏障被破坏^[42]^。神经肽P物质（neuropeptide substance P, SP）也与血脑屏障破坏相关，乳腺癌细胞分泌的SP可以调节细胞粘附并促进肿瘤细胞迁移到BMEC。SP可以激活BMEC产生TNF-α和Ang-2增加血脑屏障通透性，动物实验中SP抑制剂可以明显减少肿瘤细胞在大脑的定植^[43]^。Sevenich等^[44]^最近研究发现巨噬细胞和肿瘤细胞都能够产生组织蛋白酶S（Cathepsin S），它能通过水解连接粘附分子JAM-B特异性调节血脑屏障的迁移。药物抑制Cathepsin S表达可以减少脑转移的发生。因此恢复血脑屏障的完整性可抑制脑转移的发生和发展。

#### 2.4.3 肿瘤细胞穿透血脑屏障后增殖的分子机制

肿瘤细胞穿透血脑屏障后大部分细胞死亡，存活的细胞可与毛细血管表面粘附并围绕毛细血管生长，其机制尚未完全阐明。有研究^[45]^表明纤维蛋白溶酶可通过两种途径抑制癌细胞的转移，一是将星形胶质细胞中凋亡相关因子配体FasL转换为癌细胞旁分泌死亡信号途径，或者通过抑制轴突探路分子L1细胞粘附分子（L1 cell adhesion molecular, L1CAM）起作用。而脑转移灶中丝氨酸蛋白酶抑制剂（serine protease inhibitors, Serpins）可抑制纤维蛋白溶酶的生成并通过保护癌细胞逃避死亡生物程序及促进血管的共选择作用保证脑转移灶中癌细胞的存活。Woditschka等^[46]^发现乳腺癌脑转移灶中乳腺癌1号基因环状结构域1（BRCA1 associated RING domain 1, BARD1）和DNA修复基因RAD51过表达，将过表达BARD1或RAD51的细胞注入鼠尾静脉仅出现脑转移而未形成肺转移灶，体外实验中过表达BARD1和RAD51的细胞可干扰DNA损伤修复，从而诱导细胞生长和克隆增殖，其过表达与微环境中活性氧诱导的内源性基因毒性应激相关，应用氧自由基清除剂可以抑制BRAD1和RAD51诱导的细胞增殖及转移，提示研究脑转移灶细胞增殖的分子机制有利于开发小分子抑制剂治疗脑转移^[47]^。

## 3 展望

从传统的放化疗到分子靶向治疗，原发肿瘤的治疗已经取得了很大的进展，而转移瘤的治疗是目前研究的一大难题。肿瘤微环境不但影响肿瘤转移的发生和发展，还会影响原发肿瘤的治疗疗效。虽然目前有很多临床前研究和临床实验陆续发现和验证微环境中的治疗靶点，并取得一定的进展，但由于肿瘤微环境复杂多变，异质性极高，仍有很多机制不明。推动微环境的研究工作仍有很多问题亟需解决：如何更精确的模拟体内微环境？如何发现肿瘤微环境独有的特征？如何早期识别微转移灶，将转移的苗头扼杀在萌芽之中？如何避免靶向肿瘤微环境药物的毒副作用？肿瘤的治疗疗效判断目前有RECIST标准作为指导，那么又该如何评价靶向微环境治疗的疗效？如何根据肿瘤转移的时间特异性和空间特异性进行合理的治疗？如何有效利用针对微环境的治疗降低肿瘤复发的风险？靶向药物治疗中会出现耐药，那么微环境中的治疗靶点会不会在治疗压力下发生表观遗传学的改变？总之，肿瘤微环境的研究成功将是肿瘤治疗的另一大突破，解决每一个难题都将为攻克癌症提供新视角。
